# Comparison of base excess, lactate and pH predicting 72-h mortality of multiple trauma

**DOI:** 10.1186/s12873-021-00465-9

**Published:** 2021-07-07

**Authors:** Junfang Qi, Long Bao, Peng Yang, Du Chen

**Affiliations:** 1 Division of Emergency Medicine, the First Affiliated Hospital of Soochow University, Suzhou, China; 2 Division of Critical Care Medicine, the First Affiliated Hospital of Soochow University, Suzhou, China

**Keywords:** Blood gas analysis, Base excess, Lactate, pH, Multiple trauma, Mortality, Predictive value

## Abstract

**Objective:**

To compare the predictive values of base excess (BE), lactate and pH of admission arterial blood gas for 72-h mortality in patients with multiple trauma.

**Methods:**

This was a secondary analysis based on a publicly shared trauma dataset from the Dryad database, which provided the clinical data of 3669 multiple trauma patients with ISS > = 16. The records of BE, lactate, pH and 72-h prognosis data without missing values were selected from this dataset and 2441 individuals were enrolled in the study. Logistic regression model was performed to calculate the odds ratios (ORs) of variables. Area under the curve (AUC) of receiver operating curve (ROC) was utilized to evaluate the predictive value of predictors for 72 h in-hospital mortality. Pairwise comparison of AUCs was performed using the Delong’s test.

**Results:**

The statistically significant correlations were observed between BE and lactate (*r* = − 0.5861, *p* < 0.05), lactate and pH (*r* = − 0.5039, *p* < 0.05), and BE and pH (*r* = − 0.7433, *p* < 0.05). The adjusted ORs of BE, lactate and pH for 72-h mortality with the adjustment for factors including gender, age, ISS category were 0.872 (95%CI: 0.854–0.890), 1.353 (95%CI: 1.296–1.413) and 0.007 (95%CI: 0.003–0.016), respectively. The AUCs of BE, lactate and pH were 0.693 (95%CI: 0.675–0.712), 0.715 (95%CI: 0.697–0.733), 0.670 (95%CI: 0.651–0.689), respectively.

**Conclusions:**

There are significant correlations between BE, lactate and pH of the admission blood gas, all of them are independent predictors of 72-h mortality for multiple trauma. Lactate may have the best predictive value, followed by BE, and finally pH.

## Background

Trauma is a public health problem of widespread concern [1], especially multiple trauma. Patients with multiple trauma often indicates that they are seriously injured, in a rapidly changing condition and have a higher risker of death, which means that rapid and accurate assessment is very essential and crucial. Early screening of patients with in-hospital mortality risk is very important for rational allocation of medical resources, ensuring patient safety and reducing medical risks [2], which requires researchers to conduct in-depth study on the prognostic indicators of patients with multiple trauma.

Blood gas analysis is of great value in evaluating the condition and prognosis of critically ill patients, as well as in patients with multiple trauma [3–5]. BE, lactate and pH in blood gas could reflect the internal environment of patients and trauma patients are often accompanied by significant changes in the above-mentioned parameters [5–8]. Previous studies have shown that the three indicators could evaluate the condition and predict the prognosis [3, 9, 10], but there are few comparative studies on the predictive values of these markers in trauma population. Therefore, the current study compared the predictive values of the three parameters for 72-h mortality in patients with multiple trauma to identify which parameter can better predict the prognosis of polytrauma patients.

## Methods

### Patients and data extraction

This was a retrospective study based on a publicly shared trauma dataset from the Dryad database [11]. The Dryad is a open resource that makes research data discoverable, freely reusable, and citable, which provides a general-purpose home for a wide diversity of data types. It provides a large number of datasets in which patient information is anonymous. The data collection has been approved by the local ethics committee and conforms to the principles outlined in the Declaration of Helsinki. There was article that had been published on this dataset [12, 13], and our research was a secondary analysis of the dataset. This dataset includes multiple injured patients treated at a Level 1 trauma center of the University Hospital Zurich from January 1, 1996 to January 1, 2013. The inclusion criteria were: adult patients, treated due to polytrauma at one Level 1 trauma center, and an admission time of less than 24 h after the trauma. Patients with oncological diseases, chronic diseases, and genetic disorders that affect the musculoskeletal system were excluded.

The records of BE, lactate, pH and 72-h prognosis data without missing values were selected from this dataset. Finally, 2441 individuals were enrolled in our study. Patients’ characteristics, including sex, age, admission ISS score, three parameters of admission blood gas analysis (BE, lactate, pH) and the 72-h mortality were recorded in the dataset and included in the statistical analysis.

### Statistical analysis

The distributions of the continuous and categorical indicators were described as median (IQR) and frequency/percentages, respectively. Pearson chi square test was used to assess for statistical significance for the categorical variables, while the Mann-Whitney U test were used for the continuous indictors. The correlation between variables was demonstrated by scatter plot and the Spearman correlation analysis. Logistic regression model was performed to calculate the odds ratios (ORs) of variables. Receiver operating curve (ROC) was utilized to evaluate the predictive values of predictors for 72 h in-hospital mortality. Pairwise comparison of AUCs was performed using the Delong’s test. Statistical analyses were performed by STATA 15 and MedCalc 15. The tests with *P* < 0.05 were interpreted as a significant difference. The *P* values of multiple comparison of AUCs were adjusted by Bonferroni correction and tests with *P* < 0.017 were interpreted as a significant difference.

## Results

**Fig. 1:**
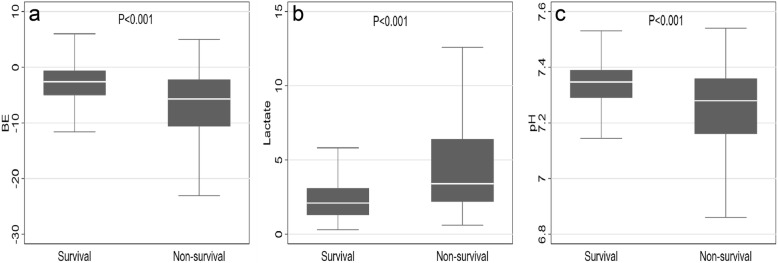
Box and whisker plots of BE, lactate and pH

A total of 2441 patients were evaluated in the study, including 1827 males and 614 females, 1946 (79.72%) in the survival group and 495 (20.28%) in the non-survival group. Following dichotomization, patients grouped according to survival and death in 72-h were significantly different with respect to age, ISS category, BE, lactate, and pH (*P* < 0.05). Compared with those of survivors, patients in non-survival group were older (*P* < 0.001) and showed a higher lactate (*P* < 0.001), a worse BE (*P* < 0.0001), and a lower pH (*P* < 0.001) (Table 1 and Fig. 1).

**Table 1:** Baseline characteristics

Variables	Total 2441 (100.00)	Survival 1946 (79.72)	Non-survival 495 (20.28)	P
**Gender n(%)**				0.271
Female	614 (25.15)	480 (24.67)	134 (27.07)	
Male	1827 (74.85)	1466 (75.33)	361 (72.93)	
**Age (year)**	42 (32)	41 (30)	49 (39)	< 0.001
**ISS**	26 (18)	25 (17)	34 (25)	< 0.001
**ISS category n(%)**		827 (42.50)		< 0.001
ISS < 25	890 (36.46)	827 (42.50)	63 (12.73)	
ISS ≥ 25	1551 (63.54)	1119 (57.50)	432 (87.27)	
**BE (mmol/L)**	−3.00 (5.10)	−2.6 (4.4)	−5.7 (8.4)	< 0.001
**Lactate (mmol/L)**	2.30 (2.13)	2.1 (1.8)	3.4 (4.2)	< 0.001
**pH**	7.34 (0.12)	7.35 (0.10)	7.28 (0.20)	< 0.001

ISS, revised trauma score; BE, base excess.

**Fig. 2:**
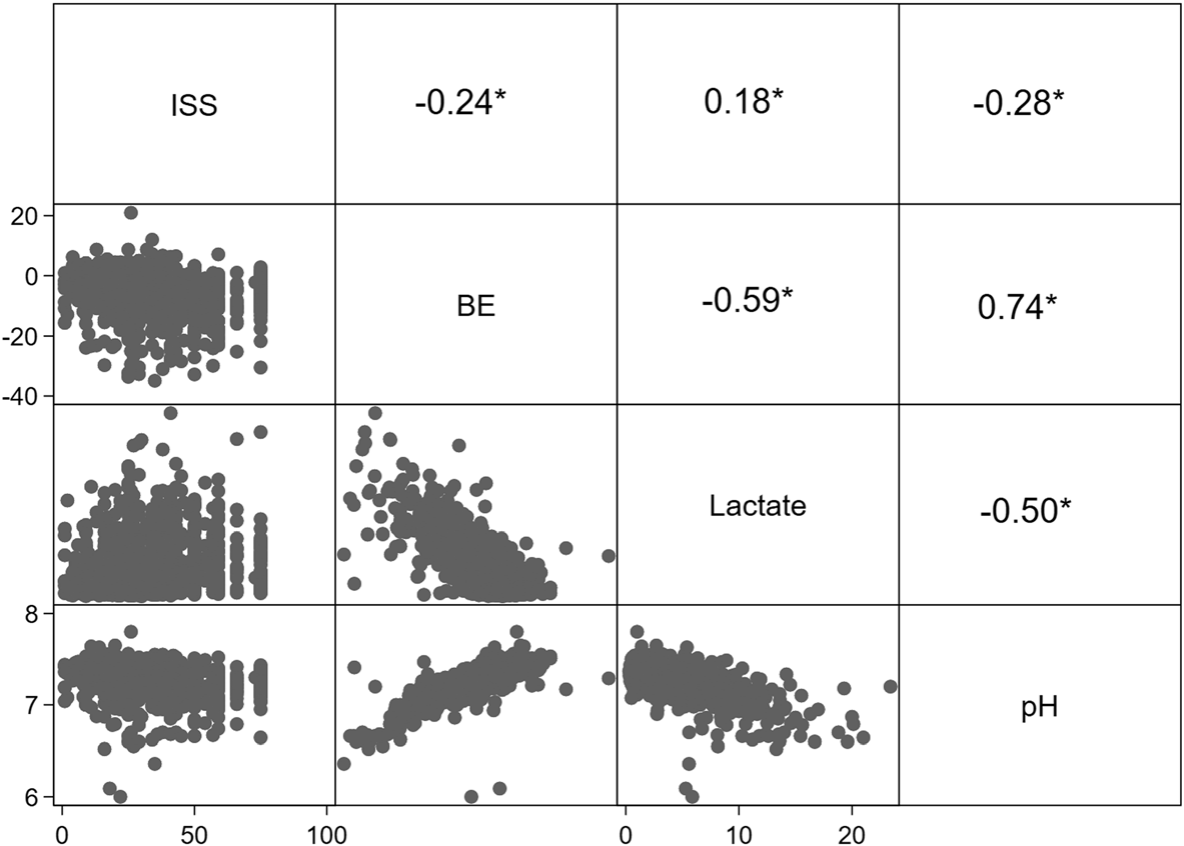
Scatter plots and correlation coefficients of ISS, BE, lactate and pH (* *P* < 0.05)

Figure 2 demonstrated strong correlations between BE and lactate (*r* = − 0.5861, *p* < 0.05), lactate and pH (*r* = − 0.5039, *p* < 0.05), BE and pH (*r* = − 0.7433, *p* < 0.05), while weak associations were also observed between the three variables (BE, lactate and pH) and ISS at admission.

Univariate logistic regression analysis revealed that 72-h mortality of multiple trauma was closely related to BE, lactate, pH (*P* < 0.001). The crude ORs of BE, lactate, pH were 0.886 (95%CI: 0.849–0.884), 1.353 (95%CI: 1.229–1.410) and 0.005 (95%CI: 0.002–0.011), respectively. Univariate logistic regression analysis also revealed that 72-h mortality was related to age and ISS category (*P* < 0.001). The ORs associated with gender, age and ISS category were 0.822 (95% CI: 0.707–1.103, *P* = 0.274), 1.018 (95% CI: 1.013–1.023, *P* < 0.001) and 5.068 (95% CI: 3.833–6.700, *P* < 0.001).

A multivariable logistic model was constructed to determine the association between BE, lactate, pH and 72-h mortality adjusted for gender, age and ISS category. The adjusted ORs of BE, lactate and pH were 0.872 (95%CI: 0.854–0.890), 1.353 (95%CI: 1.296–1.413) and 0.007 (95%CI: 0.003–0.016), respectively. (Table 2). BE, lactate and pH are independent predictors of mortality, which suggests that for an increase of one unit of lactate, the risk of an unfavorable prognosis was raised by 35.3%, for a decrease of one unit of BE, the risk of an unfavorable prognosis was raised by 12.8% and for a decrease of one unit of pH, the risk of an unfavorable prognosis was raised by 99.3%.

**Table 2:** Logistic regression analyses

Variables	Univariate			Multivariate		
	OR	95%CI	P	OR	95%CI	P
**BE**	0.866	0.849–0.884	< 0.001	0.872	0.854–0.890	< 0.001
**Lactate**	1.353	1.299–1.410	< 0.001	1.353	1.296–1.413	< 0.001
**pH**	0.005	0.002–0.011	< 0.001	0.007	0.003–0.016	< 0.001

Variables in multivariate logistic models were adjusted for gender, age and ISS category.

**Fig. 3:**
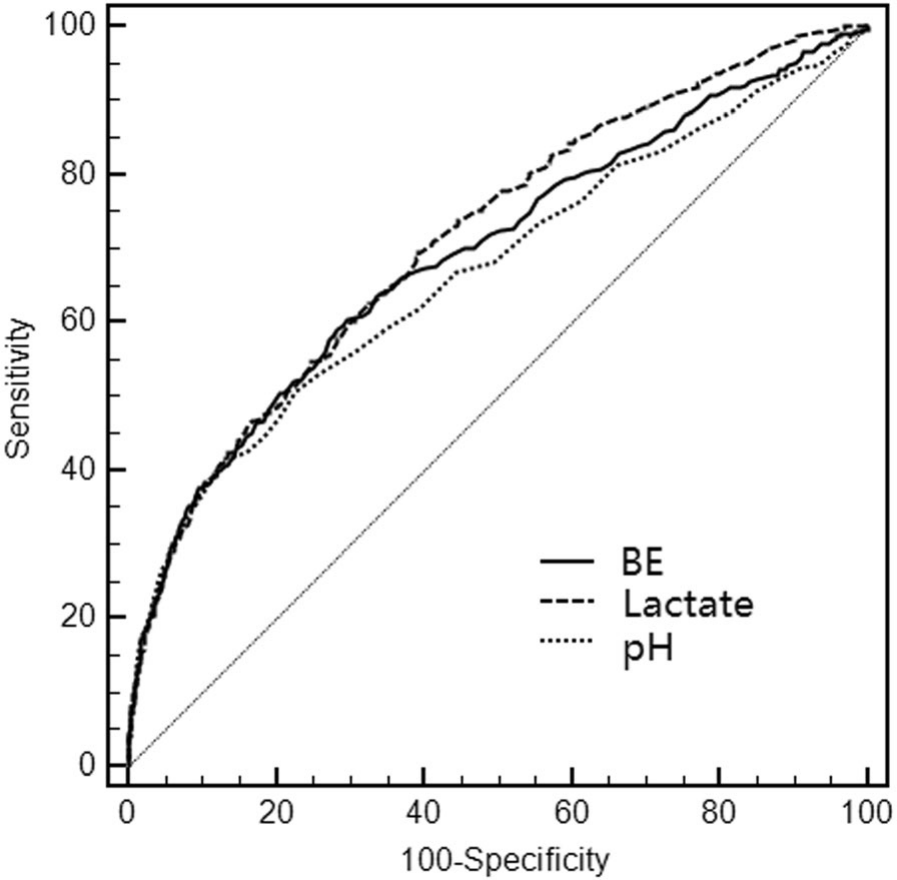
ROC curves of BE, lactate and pH for 72 h in-hospital mortality of multiple trauma

To compare the predictive value of the BE, lactate and pH for 72-h mortality of multiple trauma patients, ROC curve was plotted (Fig. 3). The AUCs of BE, lactate and pH were 0.693 (95%CI: 0.675–0.712), 0.715 (95%CI: 0.697–0.733), 0.670 (95%CI: 0.651–0.689), respectively. By pairing and comparing the AUCs, we found that there was no statistical difference between lactate and BE (*P* = 0.068), between pH and BE (*P* = 0.020), while the difference pH and lactate (*P* < 0.001) were statistically significant (Table 3).

**Table 3:** ROC curve analyses

Variable	AUC	SE	95%CI	threshold values	sensitivity	95%CI	specificity	95%CI	positive likelihood ratios	95%CI	negative likelihood ratios	95%CI
**BE**^**a**^	0.693	0.0143	0.675–0.712	≤ − 4.6	59.19	54.7–63.6	71.84	69.8–73.8	2.10	1.9–2.3	0.57	0.5–0.6
**Lactate**^**b**^	0.715	0.0132	0.697–0.733	> 2.42	69.49	65.2–73.5	60.74	58.5–62.9	1.77	1.6–1.9	0.50	0.4–0.6
**pH**^**c**^	0.670	0.0149	0.651–0.689	≤7.24	41.82	37.4–46.3	87.00	85.4–88.5	3.22	2.8–3.8	0.67	0.6–0.7

Pairwise comparison of AUCs: a vs b: *P* = 0.068; a vs c: *P* = 0.020; b vs c: *P* < 0.001.

AUC: area under the curve; SE: standard error; CI: confidence interval.

## Discussion

Consistent with previous studies, we found that BE, lactate and pH could to some extent reflect the severity and predict the risk of death in patients with multiple trauma. Meanwhile, our study indicated that for the 72-h mortality of multiple trauma, the predictive value order of the three parameters was: lactate > BE > pH.

Base excess refers to the amount of acid needed to titrate 1 L blood to normal pH (7.40) under standard conditions of normal PaO2, PaCO2 and 37.0 °C. The normal range of base excess is − 2.0 mmol ~ + 2 mmol/L. BE, a calculated value based on bicarbonate and pH, is an indirect estimate of tissue acidosis caused by tissue perfusion damage [14–16]. Admission BE is a well-recognized injury marker that could assess the severity of trauma and predict post-traumatic outcome events [3]. There were many studies showed that initially negative BE could predict the risk of death in trauma patients, which means that the worse the BE, the higher the hospital mortality [17–19]. In these studies, we could observe that the average BE of survivors tends to be higher than that of dead patients, which is also reflected in our study (− 2.6(4.4) vs − 5.7(8.4), *P* < 0.001). We further explored the association between BE and 72-h mortality of multiple trauma by establishing logistic regression model and observed that BE was an independent predictor of 72-h mortality in patients with multiple trauma. The OR was 0.872 (95%CI: 0.854–0.890), indicating that each mmol/l drop in BE increased the risk of death by 12.8%. Similarly, The study of Lichtveld et al. [18] found that BE was an independent factor predicting death occurring in trauma patients and the OR was 0.92 (95%CI: 0.89–0.95), suggesting that for a decrease of one unit of BE, the risk of mortality was raised by 8%. Our findings were consistent with previously published studies that suggested that poor BE was associated with adverse outcomes and indicated a higher risk of death in multiple trauma patients. Area under the curve (AUC) of receiver operating curve (ROC) was utilized to evaluate the predictive value of BE for 72-h mortality and the AUC was 0.693(95%CI:0.675 ~ 0.712; *P* < 0.001), which was lower than 0.856 reported by ABT [15].

Lactate is a common biomarker for diagnosis of shock and monitoring of resuscitation in clinic, which shows the value not only in patients with sepsis [20], but also in patients with trauma. Although lactate clearance was confirmed to be significantly associated with poor prognosis and was reliable indicator of post-traumatic mortality, surprisingly few studies explored the predictive value of initial lactate [9]. It is worth mentioning that admission lactate is important as an early sign of metabolic disorders. Although continuous lactate measurements are optimal as part of resuscitation, it is undeniable that admission lactate is a result that we get earlier, and that highly elevated admission lactate levels could remind clinicians to pay more attention to these patients with abnormal lactate level, guide them to perform greater scrutiny, give more aggressive resuscitation, or earlier surgical intervention. In current study, it was observed that admission lactate level of non-survivors was significantly higher than that of survivors (3.4 vs 2.1 mmol/l), which was similar with the study conducted by Gale [21] and the research performed by Abt [15]. A multivariable logistic regression model was constructed to determine the association between lactate and 72-h mortality and observed that admission lactate was an independent predictor of 72-h mortality in patients with multiple trauma. The OR was 1.353 and was slightly higher than 1.01 [22], 1.21 [23], 1.20 [24] that provided by previous studies, suggesting that the higher the level of lactate, the greater the risk of mortality. Area under the curve (AUC) of receiver operating curve (ROC) was utilized to evaluate the predictive value of lactate for 72-h mortality and the AUC was 0.715, which was similar to 0.716 reported by Sammour [25] and lower than 0.78 reported by Régnier [26].

pH is a direct measurement corresponding to low perfusion, reflecting the collective metabolic effects of oxygen debt, metabolic buffer and compensatory respiration [6]. Clinically, although the biochemical markers such as BE and lactate may be discussed more frequently, they could not capture acidosis from other sources, such as concomitant respiratory failure. This is why, despite the use of alternatives, pH is still essential in clinic, which still has its own unique value in reminding doctors how serious a patient’s condition is [10]. The literature on the prognostic value of pH in trauma patients is limited. A previous study conducted by Abt [15] indicated that pH could discriminate early survivors from non-survivors with severe pelvic trauma and hemorrhagic shock and a recent study conducted by Ross [10] reported that initial pH was an independent predictor of in-hospital mortality, and the odds of death increases with the decrease of pH. The latter study showed that the patients with pH < 7.0 had six times the odds of death, compared with those patients with pH > 7.0. However, the inclusion criteria of latter study were special, which stipulated that only patients with a venous blood gas presentation pH < 7.30 were included in the analysis. It is not difficult to observe that the participants included in the above two studies were in an extremely bad condition and they tended to be seriously injured, which limited the applicability of their conclusions in traumatic people.

As suggested by previous studies, BE, lactate and pH were biomarkers of prognosis in trauma patients and had values in predicting mortality in trauma population. In addition, Our findings indicated that there were strong correlations between the three variables. The research showed that there was a strong correlation between BE and lactate, (*r* = − 0.59, *P* < 0.05), which was demonstrated in animal experiments and was similar with a clinical research [27]. Although it was known that the results of these three parameters reflected the degree of shock and low perfusion of patients and could predict the mortality of patients with trauma, there was still controversy as to which initial measurement is more valuable in clinic [6, 21, 27–29]. There were some studies that evaluated and compared the prognostic value of initial BE and lactate respectively. Davis’s research showed BE categories discriminated high risk trauma patients better than lactate [27], while Gale demonstrated that initial lactate better predicts in-hospital mortality than initial BE [21]. The end-point observation events of both studies were similar and set to the 24-h outcome, but there are differences in population inclusion and methodology between the two studies, so the final results were not consistent. The research exploring the prognostic value of pH in trauma patients is relatively limited, and the literature comparing the value of pH with BE or lactate in trauma population is rarer. It has been reported that the changes in pH had a stronger correlation with the degree of multiple organ failure than changes in BE [6], but the end-point observation of this study was multiple organ failure and not the mortality of trauma patients explored in our research.

To the best of our knowledge, there is no definite conclusion to the comparison of the predictive value of these three variables in trauma patients. As mentioned earlier, the studies on the comparison of BE, lactate and pH to predict early mortality in trauma patients were few. In addition, these existing studies were usually small in scale and the participants were sometimes limited to specific groups of people such as patients with blunt trauma [21, 29], pelvic fractures [15], abdominal ruptures [19], macrovascular injuries [30] and so on. In current study, we included a large sample of people with polytrauma to explore and compare the predictive value of these three parameters for 72-h mortality. Our data confirmed the prognostic value of these three parameters and observed that the AUC of lactate is the largest, followed by BE, and finally pH. Overall, this is one of the few clinical studies comparing BE, lactate and pH in trauma patients upon admission. We confirmed that the three parameters were early, simple and rapid predictors of mortality in trauma patients and ranked their predictive ability, which could help clinician to rapidly carry out preliminary assessment, quickly guide triage and rationally allocates medical resources.

The research had some limitations: First, it was a study based on single-center data, the *P* value of AUC comparison between lactate and BE was 0.068, which was close to 0.05, but it had not reached the statistical difference. Further increasing the sample size may show statistical difference. Second, it was a retrospective study and the baseline characteristics of the patients were unbalanced. Although the multivariate regression model was used to adjust gender, age and ISS scores, the influence of other confounding factors could not be ruled out. Third, due to data limitations, it is not possible to analyze the correlation between the dynamic changes of the three variables and the prognosis of the patients.

## Conclusions

There are significant correlations between BE, lactate and pH of the admission blood gas, all of them are independent predictors of 72-h mortality for multiple trauma. Lactate may have the best predictive value, followed by BE, and finally pH.

## Data Availability

The datasets used and analyzed during the current study are available in Dryad database, [Halvachizadeh, Sascha (2019), Data from: How to detect a polytrauma patient at risk of complications: a validation and database analysis of four published scales, Dryad, Dataset, 10.5061/dryad.bnzs7h45v].

## Abbreviations

BE: base excess
ISS: Injury Severity Score
